# Compliance assessment and flip-angle measurement of the median nerve: sonographic tools for carpal tunnel syndrome assessment?

**DOI:** 10.1007/s00330-018-5555-3

**Published:** 2018-07-09

**Authors:** Leonhard Gruber, Marnix T. van Holsbeeck, Viviane Khoury, Christian Deml, Markus Franz Gabl, Werner Jaschke, Andrea Sabine Klauser

**Affiliations:** 1 Department of Radiology, Medical University Innsbruck, Anichstraße 35, 6020 Innsbruck, Austria; 2 Musculoskeletal Radiology, Department of Radiology, Henry Ford Hospital, 2799 W. Grand Blvd, Detroit, MI 48202 USA; 3 Division of Musculoskeletal Imaging, University of Pennsylvania Health System, 3737 Market St, Philadelphia, PA 19104 USA; 4 Department of Trauma Surgery, Medical University Innsbruck, Anichstraße 35, 6020 Innsbruck, Austria

**Keywords:** Ultrasonography, Peripheral nerves, Carpal tunnel syndrome, Diagnosis, Methods

## Abstract

**Objectives:**

To assess the diagnostic performance of median nerve (MN) flip-angle measurements, deformation during wrist flexion [transit deformation coefficient (TDC)], during compression [compression deformation coefficient (CDC)] and fascicular freedom to potentially identify fibrotic MN changes in patients with carpal tunnel syndrome (CTS).

**Methods:**

This prospective study was performed with institutional review board approval; all participants provided oral and written informed consent. Wrists in 21 healthy participants and 29 patients with CTS were examined by ultrasound. MN movement during wrist flexion, MN deformation during transition over the flexor tendons (TDC) and during controlled compression (CDC) as well as fascicular freedom were assessed. Diagnostic properties of these parameters were calculated and compared to clinical findings and cross-section area measurements (ΔCSA).

**Results:**

Low flip angles were associated with high ΔCSA at a receiver-operator characteristics area under the curve (AUC) of 0.62 (0.51-0.74). TDC [AUC, 0.83 (0.73-0.92), 76.3% (59.8-88.6%) sensitivity, 88.5% (76.6-95.7%) specificity], restricted fascicular movement [AUC, 0.86 (0.78-0.94), 89.5% (75.2-97.1%) sensitivity, 80.8% (67.5-90.4%) specificity] and compression-based CDC [AUC, 0.97 (0.94-1.00), 82.1% (66.5-92.5%) sensitivity, 94.2% (84.1-98.8%) specificity] demonstrated substantial diagnostic power (95% confidence intervals *in parentheses*).

**Conclusions:**

Fascicular mobility, TDC and CDC show substantial diagnostic power and may offer insights into the underlying pathophysiology of CTS.

**Key Points:**

*• Dynamic ultrasonography during wrist flexion and compression enables median nerve deformability assessment.*

*• Overall, reduced median nerve deformability is highly indicative of CTS.*

*• Median nerve compressibility shows higher diagnostic power than conventional cross-section area measurements.*

**Electronic supplementary material:**

The online version of this article (10.1007/s00330-018-5555-3) contains supplementary material, which is available to authorized users.

## Introduction

Carpal tunnel syndrome (CTS) is a common peripheral entrapment neuropathy with an incidence of 4-16% [1]. There is still no definitive method to establish the presence of CTS; its diagnosis still primarily relies on medical history and examination [2] as well as nerve conduction studies (NCS) [3, 4]. Sensitivity and specificity of the most common clinical signs are estimated at 50-80% and 61-92%, respectively [5], while electrodiagnostic testing is reported to possess 78-93% sensitivity and 83-87% specificity, depending on the analysis [6–8]. Some authors have reported that 25% of patients with confirmed CTS have normal electrodiagnostic findings [9], while another study found that out of 125 asymptomatic control subjects, 23 had electrophysiological median neuropathy (18%) [10].

Ultrasonography (US) is increasingly used to confirm the clinical suspicion of CTS and to determine CTS severity with parameters including differences in cross-section areas (ΔCSA) [11] and wrist-to-forearm-ratio (WFR) [12, 13]. Both have been shown to possess roughly equal diagnostic accuracy when performed at defined landmarks [11].

The pathophysiological neural changes upon repeated or sustained compression injury include oedema, demyelination and finally fibrosis [14]. Some studies have demonstrated an increase in median nerve (MN) stiffness in patients with CTS [15, 16], possibly due to an increase in carpal tunnel pressure, hypoperfusion and resultant scarring. Furthermore, strain injury due to adhesions has been suspected to affect the MN in CTS [17]. Conventional US measurements such as ΔCSA and WFR rely on an increase in MN CSA proximal to the site of compression, which is considered to reflect oedematous neural alterations. Thus, conventional US often cannot differentiate between oedema and intraneural fibrosis at a chronic stage. Accordingly, the prognostic value of US findings is still at debate and little literature on the prognostic value of US is available. Only recently have attempts been made to further quantify intraneural changes of the MN, such as by sonoelastography [15, 16] and MN deformation assessment [18].

The aims of this study were to quantify MN mobility, deformability and relative fascicular movement (“fascicular freedom”) during flexion of the wrist and external compression in healthy volunteers and patients with CTS and to evaluate their respective diagnostic value.

## Materials and methods

### Recruitment of participants

This study was performed with the approval of the ethics review board of our institution. All patients provided oral and written informed consent. Exclusion criteria were decreased range of motion of the wrist, prior trauma or surgery, a bifid MN and steroid injection into the carpal tunnel less than 3 months ago. Fifty participants were included overall in a consecutive fashion from July to September 2015. All examinations were conducted by L.G. (3 years of experience in musculoskeletal ultrasound) and A.S.K (musculoskeletal radiologist, over 10 years of experience).

### Evaluation of clinical presentation

Participants were asked a standardised four-item set of questions based on the Boston Questionnaire [19] including (1) presence of dysaesthesia or paraesthesia, (2) typical distribution pattern (first to fourth finger), (3) presence of nocturnal pain episodes and (4) weakness/clumsiness of the hands. A Tinel test was performed [20] and presence of typical sensory symptoms was noted. The final score was calculated additively with results ranging from 0 to 5 points.

### Ultrasonographic examination prerequisites and algorithm

A GE Healthcare Logic E9 (GE Healthcare, Little Chalfont, UK) with a GL15-6 MHz linear transducer was used for the ultrasound examination; participants were seated opposite the examiner with supinated wrists.

The CSA of the MN was measured at the level of the pronator quadratus muscle and at the entry of the MN into the carpal tunnel; WFR (ratio of CSAs) and ΔCSA (difference between CSAs) were calculated as described before [11, 13].

**Fig. 1:**
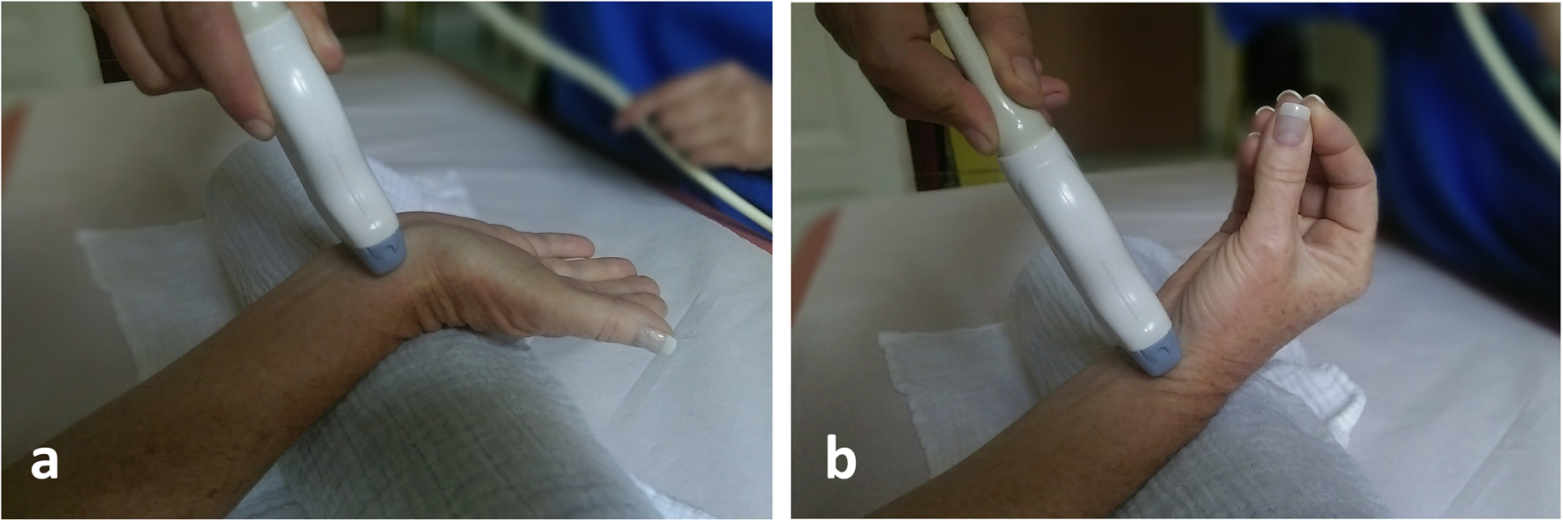
Examination procedure and findings in a healthy female volunteer (age 50 years). **a** depicts hyperextension on a standardised hypomochlion and **b** consecutive active flexion of the fingers and wrist during sonography of the median nerve (MN)

**Fig. 2:**
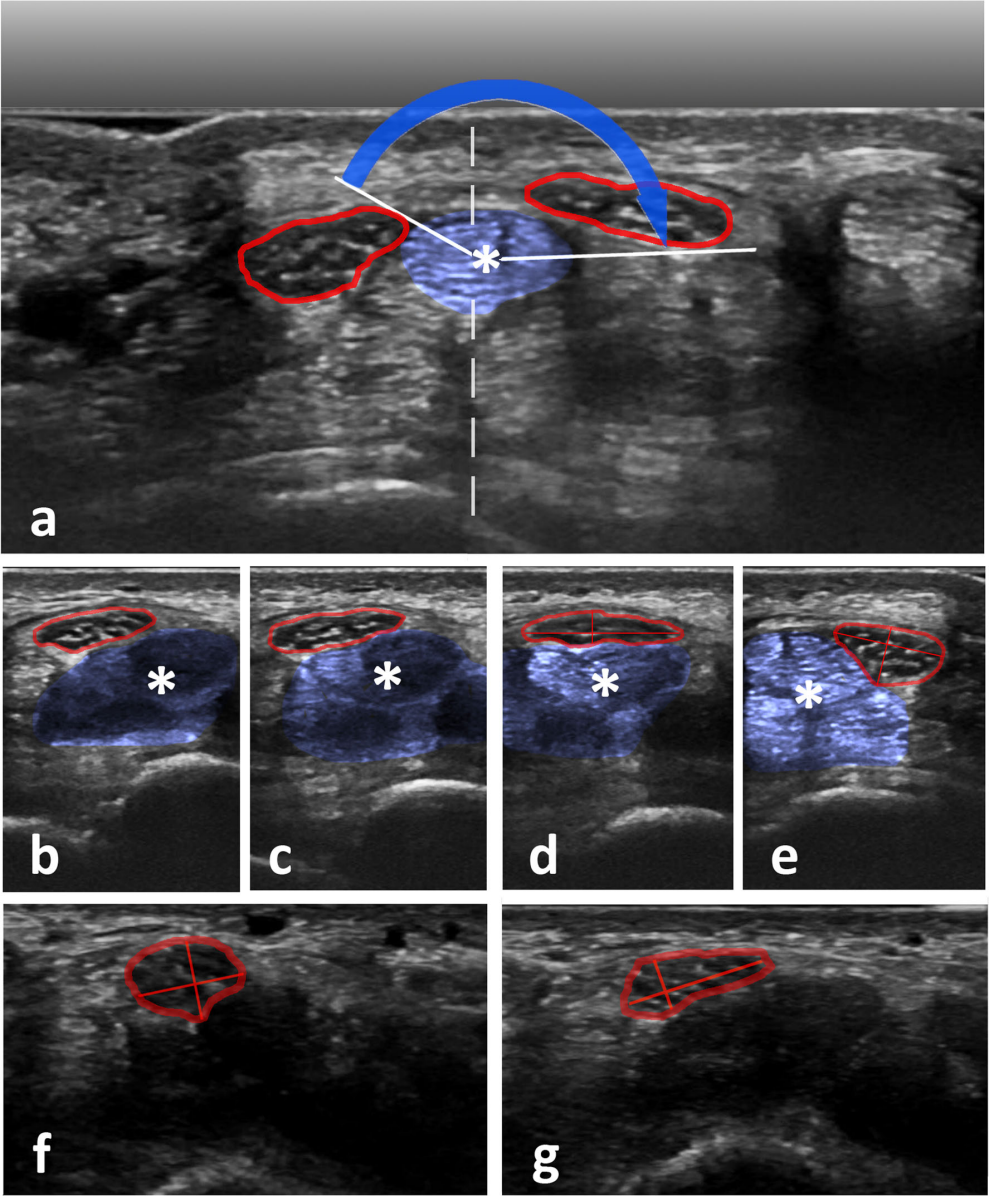
**a** Composite image taken from a cineloop (composition along the *dashed line*) before (*right*) and after transition (*left*) of the median nerve (MN) over the centre of the third flexor tendon (*blue overlay*, *) to illustrate flip-angle measurements during flexion of the wrist. The *blue arrow* marks the area covered by one predefined nerve margin of the MN (flip angle). **b-e** Configuration of the MN and relative position of its fascicles before (**b**), during (**c, d**) and after transition (**e**) over the third flexor tendon (*). *Orthogonal red lines* denote measurements of width and length of the MN CSA. **f, g** A healthy MN configuration without (**f**) and with pressure applied (**g**). Width and depth used in the calculation of the compression deformation coefficient (CDC) are given as *red lines*. This volunteer had a CDC of 2.0 with an MN aspect ratio of 0.74 before and 0.37 during compression

Participants were then instructed to rest their wrist on a standardised hypomochlion (radius, 10 cm) in supination, hyperextend their wrist and fingers (Fig. 1a) and then simultaneously bring all fingertips together and slowly flex the wrist (Fig. 1b). Maintaining the same imaging plane relative to the MN, the relative movement of the MN was recorded via cineloops during at least two cycles of flexion and extension of the wrist and fingers. The flip angle was determined as the area covered by a predefined lateral border of the MN during cineloops recorded as described above. Lines connecting a predefined lateral MN border and the centre of the third flexor tendon as a fulcrum were drawn before initiation of and after conclusion of MN movement. Finally, the angle covered between those two lines was measured (Fig. 2a).

Additionally, width and depth of the nerve CSA were measured orthogonally before initiation of movement and during movement at the site of maximum deformation and the respective aspect ratios (AR) of depth to width—similar to the ‘flattening ratio’ found in other manuscripts such as [21]—were calculated (AR_rest_ and AR_transition_) (*see* Fig. 2b-e). If not visually unequivocal, several measurements were taken to identify the site (and time point) of maximum AR_transition_. The ratio of these two ARs was then calculated to derive a transition deformation coefficient (TDC = AR_rest_/AR_transition_).

Fascicular freedom (i.e. relative movement between fascicles) was visually graded as unrestricted, moderately restricted and severely restricted (no relative movement between singular fascicles during transition) (Fig. 2b-e). Furthermore, the presence of a snapping or sudden transition of the MN was noted.

Finally, compressibility was tested via repeated controlled vertical compression and release of the wrist with the transducer at the entry into the carpal tunnel. Sufficient release was defined as filling of subcutaneous veins. Pressure was then applied until no further tissue deformation of the MN and surrounding tissue could be achieved. Width and depth of the nerve were measured with and without applied pressure and the according ARs (AR_rest_ and AR_compression_) were then used to calculate a compression deformation coefficient (CDC = AR_rest_/AR_compression_) (Fig. 2f, g) as described above for TDC.

### Statistical analysis

GraphPad Prism Pro version 6.05 (GraphPad Software, La Jolla, CA, USA) was used for statistical analysis. The *p* values were adjusted for multiple testing where appropriate. A *p* value < 0.05 was considered statistically significant. Results are given as mean ± standard deviation (SD).

Measurements of flip angles, TDC and CDC were performed in Agfa Impax EE PACS® (Agfa Healthcare, Mortsel, Belgium) after conclusion of the study and without knowledge of the participants’ symptoms or cause of referral.

Differences in gender distribution between CTS patients and controls were analysed via a χ^2^ test. Differences in age between controls and patients with CTS were calculated using an unpaired *t*-test.

Normality of distributions was assessed by D’Agostino and Pearson’s omnibus normality test. To achieve normality, a logarithmic transformation was performed for ΔCSA, WFR, TDC, and CDC values.

To illustrate the association between ΔCSA and clinical score, flip angle, TDC and CDC values (regardless of the presence of CTS), predictors were grouped by ΔCSA ranges of <3 mm^2^, 3-6 mm^2^, and >6 mm^2^. Group differences between the lowest range of normal ΔCSA values and ranges with moderate and severe increase were calculated using an ordinary one-way ANOVA with Bonferroni’s multiple comparisons test (in case of Gaussian distribution) or Kruskal-Wallis test with Dunn’s multiple comparisons test (in case of non-Gaussian distribution).

To compare the differences in predictor values between controls and patients with CTS, the same statistical procedures as described above were performed.

To assess for preliminary diagnostic values of the assessed measures, contingency tables were analysed via a two-tailed Fisher’s exact test and values for sensitivity, specificity, correct classification rate, positive predictive value (PPV) and negative predictive value (NPV) were calculated.

To assess overall diagnostic power, ROC (receiver-operating characteristics) curves were generated for clinical score, flip angle, TDC, CDC and ΔCSA. Area under the ROC curve (AUC) values of ΔCSA and CDC were compared using a weighted method proposed by Hanley et al [22].

### Sensitivity analysis

To assess potential biases introduced by the inclusion of both wrists from controls and unaffected wrists from patients with CTS, a sensitivity analysis was performed after random exclusion of one wrist in controls (*n* = 21) and exclusion of contralateral, unaffected wrists in CTS patients (*n* = 10). Resulting ROC AUC values were compared to the results from the wrist-based analysis via the methods described above by Hanley et al [22].

## Results

### Population characteristics

Twenty-nine patients with pathological electrophysiological studies and clinically proven CTS (40 symptomatic wrists) were enrolled in the study, along with 21 control patients who had no history of CTS and were referred due to other causes. Unaffected wrists of the CTS patient group were also included as controls (overall 52 asymptomatic wrists). Eight control wrists had to be excluded due to restricted range of motion (due to prior trauma, degenerative arthritis, tendinitis or Dupuytren’s disease), recent surgery or intervention. For further demographic information on study participants, please refer to Table 1.

**Table 1:** Demographic information on study participants

	Overall	CTS	Controls	*p* value
Age [years]	56.1 ± 17.6 (24–95)	59.5 ± 17.0 (28–95)	53.6 ± 17.9 (24–81)	0.11
Male gender [%]	28.0	17.2	42.9	0.047

Group characteristics of all study participants, patients with CTS and control participants

Values given as mean ± standard deviation (SD), ranges *in brackets*; *p* values denote significance levels for the comparison between CTS patients and controls

For the following wrist-based analyses, the control group consisted of both wrists in 21 controls without CTS [nine men (mean age, 40.4 years; age range, 24-81 years); 12 women (mean age, 57.6 years; age range, 44-76 years)] and unaffected wrists in patients (*n* = 10). The patient group consisted of 40 wrists in 29 patients with CTS [five men (mean age, 73.9 years; age range, 55-95 years); 24 women (mean age, 57.5 years; age range, 28-89 years)].

### Association of MN swelling and clinical score, flip angle, TDC and CDC

**Fig. 3:**
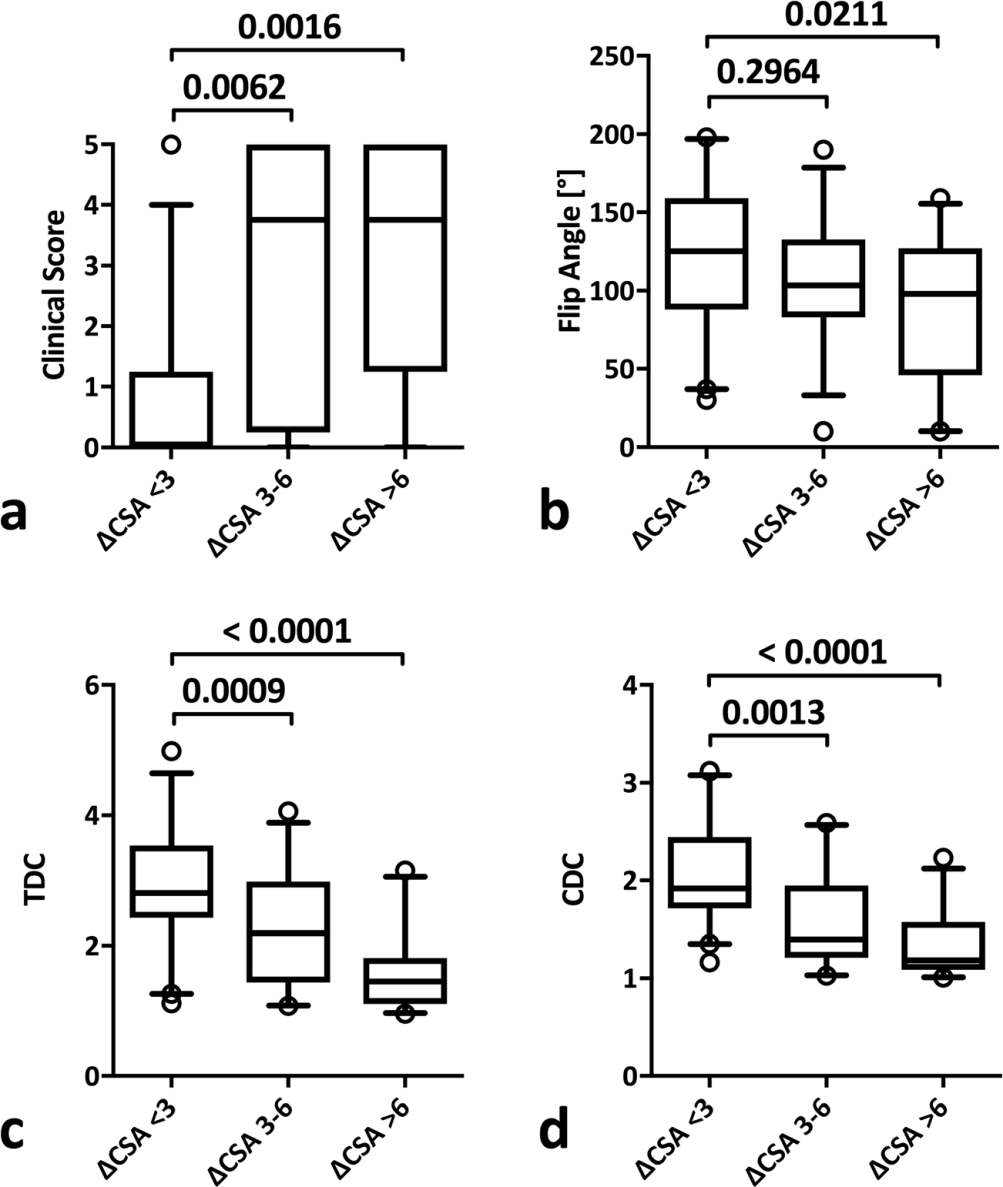
Box plots of the simplified clinical score (**a**), flip angle (**b**), transit deformation coefficient (TDC) (**c**) and compression deformation coefficient (CDC) (**d**) according to ΔCSA categories

Higher MN ΔCSA was associated with an increase in clinical severity as measured by the clinical score (ΔCSA <3 mm^2^, 0.85 ± 1.29 items; 3-6 mm^2^, 2.90 ± 2.03 items; >6 mm^2^, 3.25 ± 1.96 items; *p* = 0.0003 and < 0.0001, respectively) (Fig. 3a), while only a substantial swelling of the MN was associated with a reduced flip angle (<3 mm^2^, 122.8 ± 45.4°; 3-6 mm^2^, 107.5 ± 36.8°; >6 mm^2^, 89.9 ± 44.2°; *p* = 0.269 and 0.021, respectively) (Fig. 3b).

On the other hand, there was a significant decrease in TDC (ΔCSA <3 mm^2^, 2.98 ± 0.94; 3-6 mm^2^, 2.23 ± 0.86; >6 mm^2^, 1.57 ± 0.54; *p* = 0.0009 and <0.0001, respectively) (Fig. 3c) and CDC (ΔCSA <3 mm^2^, 2.05 ± 0.49; 3-6 mm^2^, 1.60 ± 0.49; >6 mm^2^, 1.31 ± 0.31; *p* = 0.0013 and <0.0001, respectively) (Fig. 3d) for both ranges.

### Preliminary diagnostic performance of predictors

Overall diagnostic performance of clinical scoring was good at a cut-off of two out of five items (AUC, 0.939; Table 2, Fig. 5a). Controls scored below one item on average (0.73 items; 95% CI, 0.4–1.0 items), while patients with CTS scored four out of five items on average (95% CI, 3.5–4.4 items).

**Table 2:** Diagnostic properties of the examined predictors

	AUC	*p* value	Cut-off	Sensitivity [%]	Specificity [%]	PPV	NPV	Correct classification rate [%]
Clinical score	0.94 [0.89–0.99]	<0.0001	40% (2 items)	80.0 [64.4–91.0]	94.2 [84.1–98.8]	91.4 [76.9–98.2]	86.0 [74.2–93.7]	88.0
ΔCSA	0.88 [0.80–0.95]	<0.0001	4	80.0 [64.4–91.0]	86.5 [74.2–94.4]	82.1 [66.5–92.5]	84.9 [72.4– 93.3]	83.7
WFR	0.86 [0.78–0.94]	<0.0001	1.4	87.5 [73.2–95.8]	78.9 [65.3–88.9]	76.1 [61.2–87.4]	89.1 [76.4–96.4]	82.6
Flip angle	0.62 [0.51–0.74]	0.02	118°	68.4 [51.4–82.5]	57.7 [43.2–71.3]	54.2 [39.2–68.6]	71.4 [55.4–84.3]	62.2
TDC	0.83 [0.73–0.92]	<0.0001	2.0	76.3 [59.8–88.6]	88.5 [76.6–95.7]	82.9 [66.4–93.4]	83.6 [71.2–92.2]	83.3
CDC	0.97 [0.94–1.00]	<0.0001	1.5	82.1 [66.5–92.5]	94.2 [84.1–98.8]	91.4 [76.9–98.2]	87.5 [75.9–94.8]	89.0
Fascicular freedom	0.86 [0.78–0.94]	<0.0001	Severe restriction	89.5 [75.2–97.1]	80.8 [67.5–90.4]	77.3 [62.2–88.5]	91.3 [79.2–97.6]	84.4

Diagnostic properties of tests based on clinical score, ΔCSA, WFR, flip-angle, TDC, CDC and fascicular freedom; 95% confidence intervals are given *in square brackets*; *p* values are adjusted for multiple comparisons where appropriate

While ΔCSA and WFR showed comparable diagnostic properties (Table 2), MN flip angle measurements demonstrated a non-significant reduction in the angle covered by a predefined border of the MN in patients with CTS (*p* = 0.617) at low overall diagnostic utility (AUC, 0.625) with a sensitivity of 68.4%, specificity of 57.7%, PPV of 54.5% and NPV of 71.4% (Table 2).

Snapping MN movement during wrist movement occurred in a minority of wrists (3.3%) and, while demonstrating a high specificity for CTS (96.3%), lacked overall diagnostic power (sensitivity 26.3%, PPV 33.3% and NPV 57.5%).

Severely restricted fascicular freedom showed comparable diagnostic properties to ΔCSA and WFR [AUC, 0.86 (0.78-0.94%); sensitivity, 89.5% (75.2- 97.1); specificity, 80.8% (67.5-90.4); PPV, 77.3% (62.2-88.5); NPV, 91.3% (79.2-97.6%)] (Table 2).

**Fig. 4:**
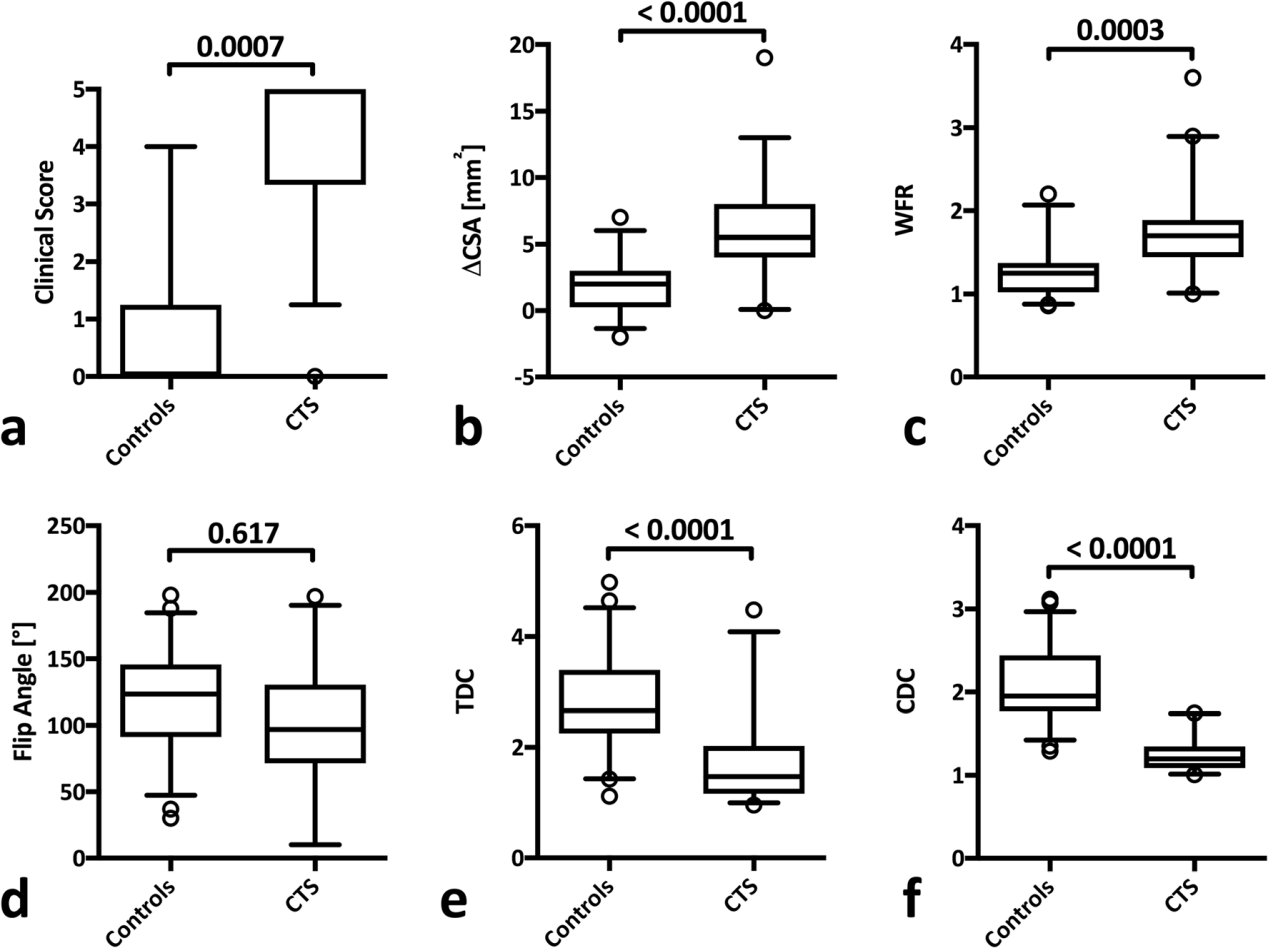
Comparison of clinical score (**a**), ΔCSA (**b**), wrist-to-forearm ratio (WFR) (**c**), flip angle (**d**), transit deformation coefficient (TDC) (**e**), and compression deformation coefficient (CDC) (**f**) between control wrists and wrists with carpal tunnel syndrome (CTS)

**Fig. 5:**
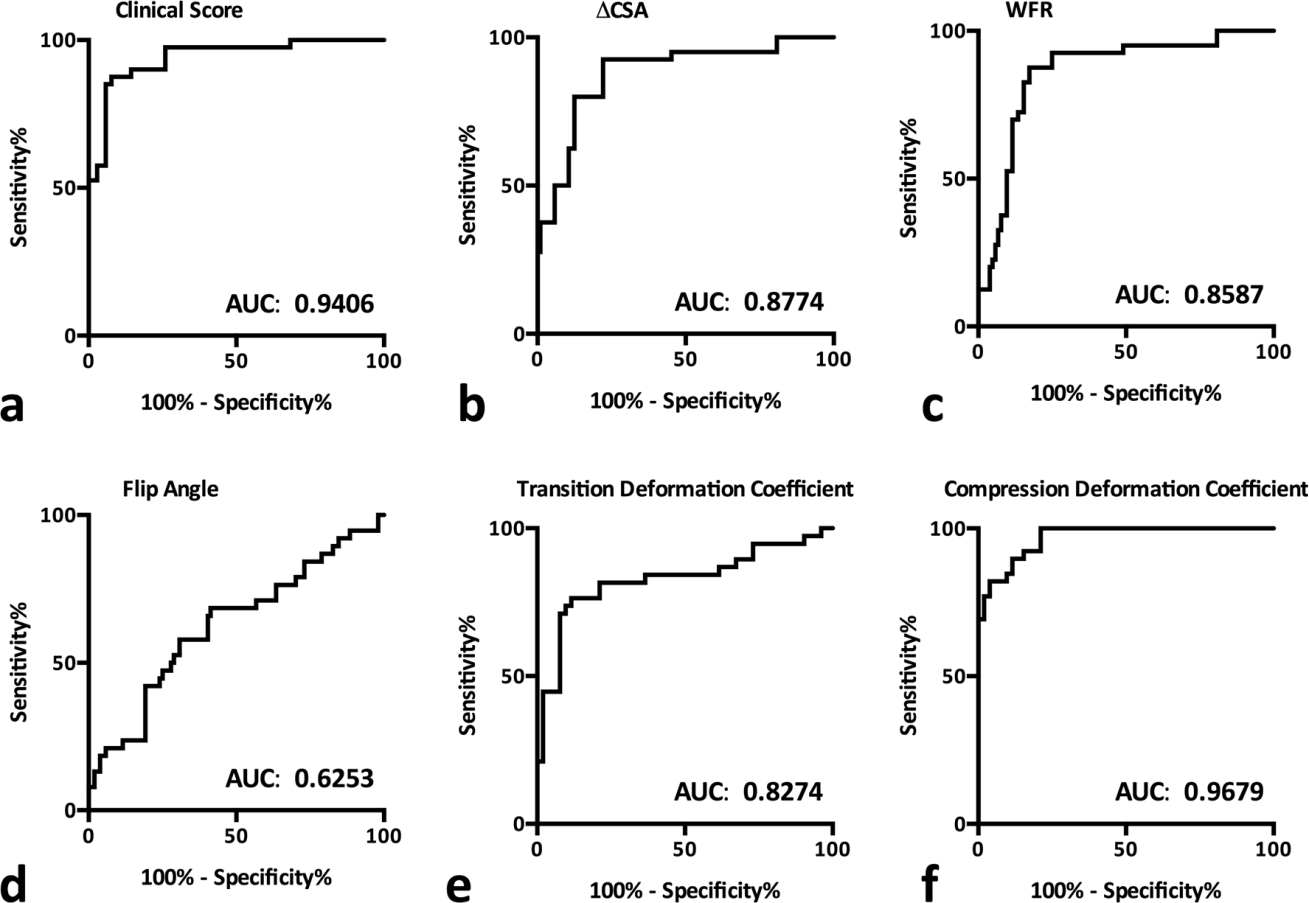
Receiver-operating characteristics (ROC) curves and corresponding area under the curve (AUC) values for clinical score (**a**), delta value (ΔCSA, **b**), wrist-to-forearm ratio (WFR, **c**), flip angle (**d**), transit deformation coefficient (TDC, **e**) and compression deformation coefficient (CDC, **f**)

MN deformability as assessed by TDC and CDC was significantly lower in wrists with CTS than in control wrists (*p* < 0.0001, respectively) (Fig. 4e/f). In regard to their respective diagnostic efficacy, TDC at a cut-off at 2.0 [AUC, 0.83 (95% CI, 0.73- 0.92)] demonstrated slightly lower diagnostic power than ΔCSA [AUC, 0.88 (95% CI, 0.80- 0.95) at a cut-off at 4 mm] and WFR [0.86 (95% CI, 0.78-0.94) at a cut-off at 1.4]. CDC at a cut-off of 1.5 showed higher values for sensitivity [82.1% (95% CI, 66.5-92.5)] and specificity [94% (95% CI, 84.1-98.8)] with a significantly higher AUC of 0.97 (95% CI, 0.94-1.00) than routine ΔCSA measurements (*p* = 0.017) (Table 2, Fig. 5e/f).

### Sensitivity analysis

A sensitivity analysis based on a patient-basis yielded comparable ROC results for all predictors with a moderate increase in ROC AUC 95% confidence interval ranges, probably due to a lower case number [clinical score, 0.94 (0.88-1.00, *p* = 0.92); ΔCSA, 0.92 (0.84-1.00, *p* = 0.41); WFR, 0.90 (0.79-1.00, *p* = 0.55); flip-angle, 0.57 (0.40-0.73, *p* = 0.57); TDC, 0.90 (0.81-0.99, *p* = 0.28); CDC, 0.98 (0.96- 1.00, *p* = 0.34)] (Appendix).

## Discussion

Diagnosis of CTS is still not following a unified approach [11, 23] and strongly relies on clinical information [3, 4]. While neurographic studies—namely nerve conduction velocity and electromyography—are well established [24], they do not offer information on the exact location or causative mechanisms of neural compression. Accordingly, US is used as a complementary assessment in patients with strong clinical suspicion of CTS. Parameters such as ΔCSA and WFR compare MN CSAs of two predefined segments of the nerve at the entry of the MN into the carpal tunnel and a predefined reference segment such as at the distal forearm [12] or over the pronator quadratus muscle [11].

Several pathophysiological mechanisms may contribute to such an increase in CSA: oedematous changes of the neural structures after repeated micro-trauma due to focal compression, shear stress and traction [23] and consequent perfusion deficits and capillary leakage [14, 24]. Nerve gliding, which is facilitated by the perineurium, epineurium and surrounding vessels [14], may also be affected. Axonal damage occurs only in later disease stages, as CTS primarily affects myelin sheaths with recurrent demyelination and remyelination [24, 25]. Studies have shown increased Schwann-cell turnover, gradual macrophage invasion and increased connective tissue content [24]. Epineural fibrosis, perineural thickening and finally demyelination may ensue after prolonged damage [14].

In accordance with previous literature [13] we could demonstrate a strong positive correlation of clinical scoring and increase in ΔCSA; no participant with a ΔCSA >8 mm scored below four out of five items. We found ΔCSA and WFR to possess diagnostic power comparable to other publications [11, 12].

Current predictors of CTS such as ΔCSA and WFR are not able to differentiate between oedematous, i.e. acute, and fibrotic, i.e. chronic, changes of the median nerve. When evaluating MN deformability, TDC, i.e. deformation during transition over the flexor tendons and especially CDC, i.e. controlled compression of the MN yielded high values for sensitivity, specificity, PPV and NPV. CDC offers slightly higher values for both sensitivity and specificity than the conventional cross-section-area-based ΔCSA method and substantially higher specificity than WFR measurements. Apart from a comparable or slightly higher correct classification rate, these predictors may also be surrogate parameters of intraneural (fibrotic) changes—even though quantification of intraneural fibrosis was not part of this study—as potential indicators for treatment stratification and outcome.

**Fig. 6:**
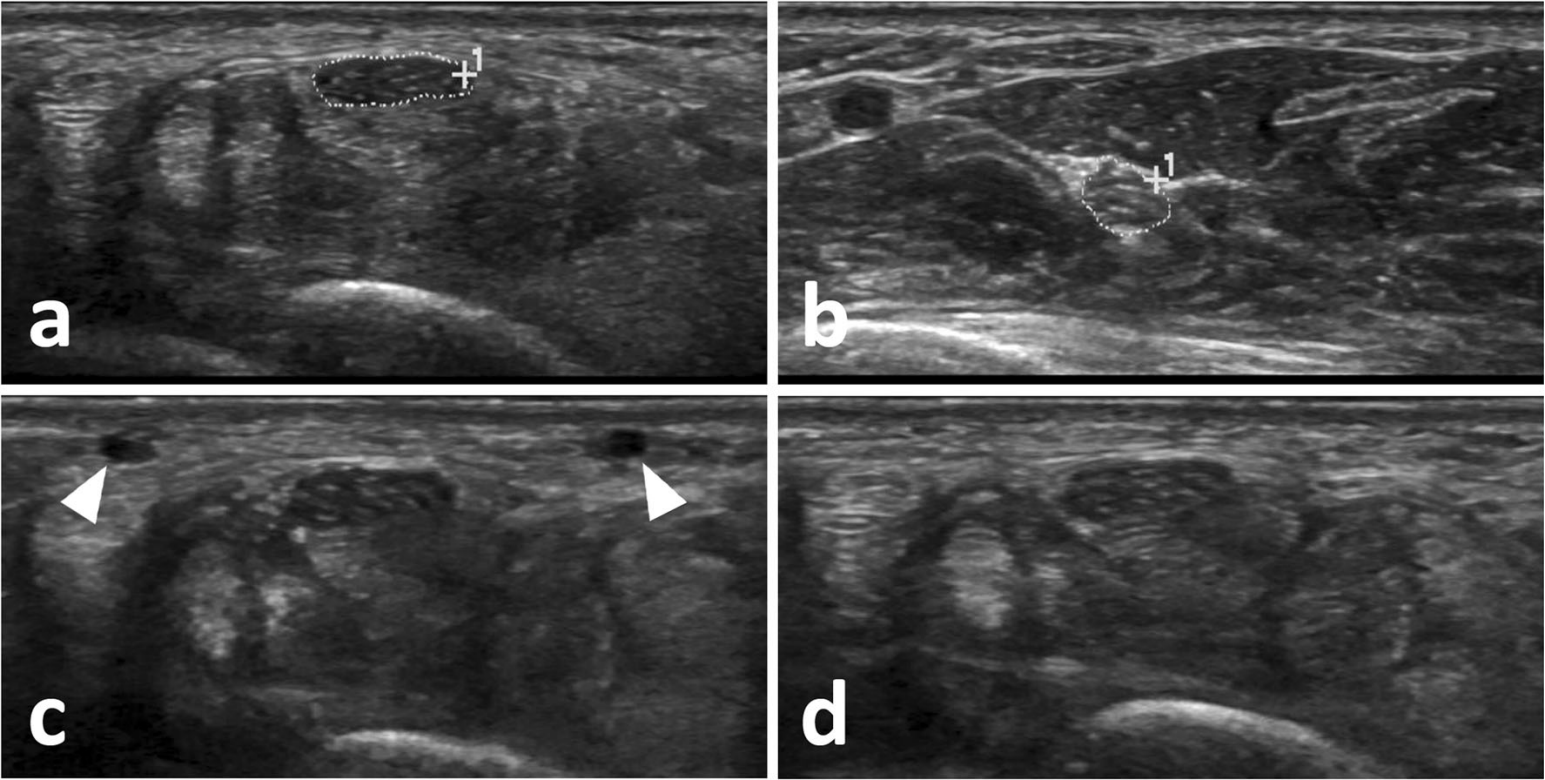
Exemplary findings in a 46-year-old woman with long-standing history of left-sided paraesthesia of the first four fingers, nocturnal pain episodes, weakness of the hand and a positive Tinel sign. The median nerve (MN) cross-section area (CSA) at the entry into the carpal tunnel was 10 mm^2^ (**a**) and 7 mm^2^ at the forearm (**b**) with a relatively low difference between CSAs (ΔCSA) of 3 mm^2^, yet a significantly reduced compressibility of the MN. **c** The MN without relevant applied pressure as can be observed by the patent superficial veins (*white arrowheads*). Controlled compression revealed no relevant deformability (**d**), yielding a compression deformation coefficient (CDC) of 1.14

Previous studies have already tried to shed light on MN [16] or carpal tunnel stiffness [15], as well as MN deformation after carpal tunnel release [18]. Similarities between our approach and elastographic methods exist: in particular, strain elastography is based on external compression and provides information on tissue deformation to derive a non-quantifiable elasticity grade [26, 27]. Our approach factors in lateral displacement of fascicles, though, and may thus prove more accurate. MN deformability measures may be influenced by an increase in carpal tunnel pressure [15, 16], and thus a reduction in CDC may not be attributable to a restriction of fascicular movement alone. Still, information on intraneural elasticity may be useful in patients with suspicion of CTS, yet normal or borderline CSA findings (exemplary Fig. 6). Such a constellation may be encountered in patients with a long-standing history of CTS and a high degree of intraneural fibrosis, for example.

By additional measurements of the flip angle—which did not provide further diagnostic information on its own—we could quantify deformation of the MN during its transition over the flexor tendons. Carpal tunnel pressure is known to be elevated in CTS due to several factors apart from MN swelling such as synovial oedema of the flexor tendons [14, 28]. While diagnostic power was lower for TDC compared to CDC, this measurement may be influenced to a lesser extent by increased carpal tunnel pressure. Exceptions may be found in patients where only extremely low flip angles can be achieved during flexion and extension of the wrist. Pathophysiological studies show that MN gliding can be restricted in CTS [14]. We observed a decrease in MN range of movement especially in severe cases of CTS with higher ΔCSA and WFR.

There are some limitations to this study. Due to consecutive patient recruitment and an overall lower incidence of CTS in men, men are under-represented in the control and patient groups. As most female patients were older than 50, hormonal influences should play a minor role, though. Furthermore, there was a higher percentage of male patients in the control group, which might have introduced a certain gender bias. No separate analysis for gender influence was carried out. Patients may not always be able to perform the flexion and extension of the wrist and fingers necessary to provoke MN displacement at the carpal tunnel. Some CTS patients described an increase of paraesthesia during wrist flexion. The measurements themselves may not be easy to achieve in every patient; the scanning plane needs to be constantly adjusted during flexion of the wrist to maintain constant perpendicular imaging plane [15], as the proximal palmar structures may displace the transducer proximally at greater flexion angles. Finally, the high diagnostic accuracy of the simplified clinical score used in this study may be an overestimation, as patients with CTS were primarily referred due to the presence of these symptoms.

Even though determination of maximum MN deformation during wrist movement was quantified by taking orthogonal measurements of width and depth and—if necessary—several measurements were taken to identify the correct site, we cannot exclude the possibility that measurements might be subject to some subjectivity. No inter-reader evaluation was performed.

Pressure application was not quantified, although filling of subcutaneous veins was deemed sufficient to define release and pressure was applied until no further deformation of the MN and surrounding tissue was observed. Some issues may arise during qualification of fascicular freedom as well, a parameter potentially prone to subjectivity. Mathematical models of relative repositioning and travel distance may alleviate this problem but seem too time-consuming for routine use. Furthermore, oedematous changes of the nerve may mask the nerve’s fascicular texture. Relative fascicular movement may be misclassified, as a lack of visibly moving fascicles may not represent a lack of mobility in such cases. From a statistical viewpoint, some bias may have been introduced by using unaffected patient wrists as controls as well—a sensitivity analysis did not alter the outcome significantly, though.

Time constraint might be another limiting factor, as a full examination of flip angle, TDC and CDC might take up to 10 min in a single wrist. For routine use it may be sufficient to just use CDC measurements and grade fascicular movement, which would only take a few minutes.

In conclusion, this study demonstrates high diagnostic accuracy of parameters of MN deformability (TDC, CDC) and fascicular freedom and their incorporation into diagnostic algorithms may further improve the ultrasound assessment in patients with suspicion of CTS or after failed carpal tunnel release as disease progress might be gradable with more accuracy.

### Electronic supplementary material

ESM 1 (MOV 64854 kb)

## Appendix: sensitivity analysis (patient-based)

**Table 3:** Sensitivity analysis results on a patient-basis (rather than on a wrist-basis) after exclusion of contralateral control wrists in CTS patients and random exclusion of one wrist in controls

	**Clinical Score**	**ΔCSA**	**WFR**	**Flip-angle**	**TDC**	**CDC**
**Sensitivity**	85.71	80.00	88.57	74.29	75.76	82.35
** 95% CI**	69.74 to 95.19	63.06 to 91.56	73.26 to 96.80	56.74 to 87.51	57.74 to 88.91	65.47 to 93.24
**Specificity**	95.45	95.45	86.36	45.45	86.36	86.36
** 95% CI**	77.16 to 99.88	77.16 to 99.88	65.09 to 97.09	24.39 to 67.79	65.09 to 97.09	65.09 to 97.09
**PPV**	96.77	96.55	91.18	68.42	89.29	90.32
** 95% CI**	83.30 to 99.92	82.24 to 99.91	76.32 to 98.14	51.35 to 82.50	71.77 to 97.73	74.25 to 97.96
**NPV**	80.77	75.00	82.61	52.63	70.37	76.00
** 95% CI**	60.65 to 93.45	55.13 to 89.31	61.22 to 95.05	28.86 to 75.55	49.82 to 86.25	54.87 to 90.64
**Likelihood Ratio**	18.86	17.6	6.495	1.362	5.556	6.039
**ROC AUC**	94.46	92.33	89.83	56.52	90.00	98.57
** 95% CI**	88.06 to 100.0	84.29 to 100.0	79.60 to 100.0	39.66 to 73.38	81.05 to 98.95	96.23 to 100.0

## Abbreviations

AR: Aspect ratio
CSA: Cross-section area
CTS: Carpal tunnel syndrome
ΔCSA: Difference in CSA measurements of the median nerve
MN: Median nerve
WFR: Wrist-to-forearm ratio
